# Bond strength between acrylic resin and artificial tooth treated with experimental silane with thio-urethane

**DOI:** 10.1590/0103-644020256136

**Published:** 2025-04-14

**Authors:** Leonardo Guimarães Sonehara, Carmem Silvia Pfeifer, Ana Paula Piovezan Fugolin, Mario Alexandre Coelho Sinhoreti, Rafael Leonardo Xediek Consani

**Affiliations:** 1 Piracicaba Dental School, State University of Campinas, SP, Brazil.; 2 Department of Restorative Dentistry, Division of Biomaterials and Biomechanics, Oregon Health and Science University, Oregon, Portland, USA.

**Keywords:** Adhesion, thio-urethane, silane, surface treatment, artificial tooth

## Abstract

This in vitro study evaluated the bond strength between thermo-activated acrylic resin and artificial tooth treated with experimental silane incorporated with thio-urethane. Artificial molar teeth were individually fixed in 50mm long cylindrical wax sticks and traditionally included in metal flasks with type III plaster coated with laboratory silicone. The tooth/wax sets were removed from the flask, the tooth separated from the wax stick, and the fitting area was cleaned with household detergent. Teeth were separated into three groups according to fitting area treatments (n=10): CON (Control, no treatment or silane application), ABR (Abrasion with diamond tip), and JAT (Blasting with 50μm aluminum oxide particles). Tooth fitting area treatment was associated with PALABOND commercial silane application (PS) or experimental silane incorporated with thio-urethane (ES). Teeth were replaced in the plaster mold, and the acrylic resin traditionally flask pressed and polymerized in a heated water bath. Tooth/resin sets were individually fixed in rigid PVC tubes with chemically activated acrylic resin, leaving a space of 1 mm between the tooth/PVC tube top, and submitted to the shear strength test in a universal test machine. Shear strength data were evaluated for normality, and subjected to one-way ANOVA and Tukey's test (5%). JAT+PS showed greater strength followed by ABR+ES. CON and JAT+ES showed lowest values and ABR+PS was intermediate. In conclusion, the artificial teeth treatments with experimental silane incorporated with thio-urethane associated with blasting or abrasion promoted different strength values when bonded to acrylic resin for denture base.

## Resumo

Este estudo *in vitro* avaliou a resistência de união entre resina acrílica termoativada e dente artificial tratado com silano experimental incorporado com tiouretano. Dentes molares artificiais foram fixados individualmente em bastões de cera cilíndricos de 50 mm de comprimento e tradicionalmente incluídos em muflas metálicas com gesso tipo III revestidos com silicone laboratorial. Os conjuntos dente/bastão de cera foram retirados da mufla, o dente separado do bastão de cera e a área de adaptação limpa com detergente doméstico. Os dentes foram separados em três grupos de acordo com os tratamentos da área de fixação (n=10): CON (Controle, sem tratamento ou aplicação de silano), ABR (Abrasão com ponta diamantada) e JAT (Jateamento com partículas de óxido de alumínio de 50μm). O tratamento da área de fixação foi associado à aplicação de silano comercial PALABOND (SP) ou silano experimental incorporado com tiouretano (SE). Os dentes foram recolocados no molde de gesso, a resina acrílica tradicionalmente prensada em mufla metálica e polimerizada em água aquecida. Os conjuntos dente/resina foram fixados individualmente em tubos de PVC rígido com resina acrílica quimicamente ativada, deixando um espaço de 1 mm entre o topo do dente/tubo de PVC e submetidos ao ensaio de resistência ao cisalhamento em máquina de ensaio universal. Os dados de resistência ao cisalhamento foram avaliados quanto à normalidade, submetidos à ANOVA um fator e ao teste de Tukey (5%). JAT+SP apresentou maior força de união seguido de ABR+SE. CON e JAT+SE apresentaram menores valores e ABR+SP foi intermediário. Conclui-se que os tratamentos de dentes artificiais com silano experimental incorporado com tio-uretano associado ao jateamento ou abrasão promoveram diferentes valores de resistência quando unidos à resina acrílica para base de prótese.

**Figure:**
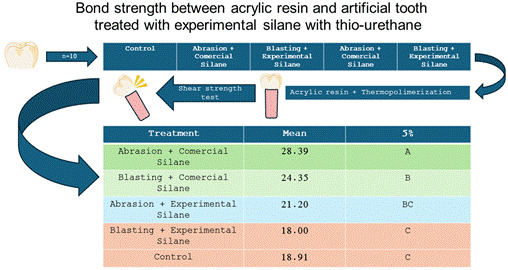

## Introduction

Dental prostheses represent indispensable value in restorative dentistry, playing an important factor in replacing missing teeth, and restoring aesthetics and chewing function in toothless patients. The careful selection of materials used in the manufacture of dental prostheses is essential to establish the durability and success of the prosthetic restorations. Frequently made with termo-activated acrylic resin, the traditional denture bases provide a series of advantages including ease of manufacture, improved aesthetics, and biocompatibility.

A previous study showed that partial or complete dentures are commonly constructed for elderly patients, and the separation of the tooth from the denture base can be frustrating for patients and also for the dentist. For this reason, research has been carried out and continues to study the issue of the bond acrylic tooth/resin of the denture base. Therefore, selecting more compatible combinations of resins for tooth and denture base can reduce the number of denture fractures and resulting repairs ^1^.

However, establishing a durable bond between artificial teeth and acrylic resin base remains a considerable challenge in the manufacture of conventional complete dentures. Failures of the bond between denture tooth/acrylic resin base have been shown to be a cause of denture tooth debonding promoting inconveniences and costly repair. The optimal combination of acrylic resin tooth, denture base material, laboratory protocol, and processing method has not yet totally been established ^2^.

The tooth/acrylic resin base bond has been considered a factor important for the stability and clinical functionality of the conventional complete denture. Consistent previous observations showed that the tooth surface contamination with wax decreased the bond strength between the tooth/denture base material ^3^. 

Additionally, most technicians do not use solvents to remove wax residue from the tooth. However, surface modification of the tooth attachment area was performed by 52% of respondents, but increased to 85% during tooth rebonding. The method of removing wax from dental prostheses with water heated to a temperature range of up to 90°C left wax residue on the tooth. However, rough surface retained more wax than smooth surface ^4^.

The bond strength between artificial tooth/denture bases can be influenced by several other factors. An earlier study showed that a stronger bond was obtained when the resin was packed late in the dough stage, and a stronger bond when high-impact resin was used. In addition, tooth surface modification by grinding or grooving did not show a significant difference compared with unmodified surfaces, and the wax-contaminated surface produced weaker bonds. On the other hand, the time of introduction and duration of water-bath processing did not promote a significant effect on bond strength, while monomer application in the tooth surface improved the bond strength, and application of resin cement produced the most significant increase in denture tooth bond strength ^5^.

Furthermore, different daily activities such as eating and chewing habits can also influence the bond strength between artificial tooth/acrylic resin base. Previous research attempted to establish an appropriate temperature range by measuring extremes of temperature that occurred orally with drinking very hot and cold liquids. The results of the study suggest that a range of zero degrees to 67°C may be appropriate to promote dental material thermocycling ^6^, probably influencing long-term tooth debonding.

Faced with these challenges, researchers have evaluated different strategies to increase the bond strength between artificial tooth/acrylic resin bases. Approaches range from creating mechanical retentions to applying monomers or adhesives to reinforce the bond between tooth/denture bases. In this sense, the previous study highlighted that ethylacetate could be an effective option in decreasing tooth/resin bond failures and also avoid repeated denture repairs ^7^. In addition, acrylic tooth bonded to three types of acrylic resin for denture base containing 10 or 20 % (vol.) cycloaliphatic comonomer incorporated into the monomer increased the shear bond strength after cyclic loading and thermal aging. However, all samples of the control and trial groups exhibited adhesive-cohesive mixed failure at the tooth/base resin interface ^8^.

Previous investigations agree regarding the benefits of creating mechanical retentions at the tooth fitting area showing a significant increase in bond strength ^9^ ^,^ ^10^ ^,^ ^11^ ^,^ ^12^ ^,^ ^13^ ^,^ ^14^, and others not reporting the same advantages ^15^ ^,^ ^16^. On the other hand, the application of adhesive or chemical agents, such as dichloromethane, resulted in a substantial increase in bond strength ^1^.

However, the latest investigations with silanes incorporated of thio-urethane showed also favorable results, indicating a potential strategy to increase the bond strength of different types of resin dental materials ^17^ ^,^ ^18^ ^,^ ^19^ ^,^ ^20^. Despite the potential benefits arising from the silanes incorporated with thio-urethane applied to the resin materials, there is still a shortage in the literature on the acrylic resin incorporated with thio-urethane. A previous study showed that, except for dimensional stability, the addition of thio-urethane to acrylic resin was not a promising method for most tested physical-chemical properties ^21^.

It has been a consensus that the mechanical retention and the application of chemical agents change the fitting area of the artificial tooth improving the bond strength with the acrylic resin for the denture base. Since the dimensional stability of the acrylic resin has not been changed with the addition of thio-urethane ^21^, it would be current and opportune to evaluate the effect of an experimental silane incorporated of thio-urethane in the strength of the artificial tooth/acrylic resin bond.

The aim of this in vitro study was to investigate the blasting or abrasion effect of the artificial tooth fitting area associated with the application of commercial silane or experimental incorporation with thio-urethane on the shear bond strength between artificial tooth/acrylic resin. The hypothesis of the study was that the experimental silane incorporated with thio-urethane would increase the bond strength between artificial tooth/acrylic resin whatever the treatment of the artificial tooth.

## Materials and methods

### Experimental design

Artificial molar teeth were subjected to two treatments on the surface of the fitting area: JAT (Blasting with 50 μm aluminum oxide particles) and ABR (Abrasion with diamond tip), and subject to the application of two silane types: PS (Palabond commercial silane) or ES (experimental silane incorporated with thio-urethane). In the control (CON), the teeth were not subjected to any type of surface treatment or silane application.

### Sample preparation

Artificial molar teeth (A1-Delara 8- 3OU; Kulzer, Sao Paulo, SP, Brazil) were divided into five experimental treatments (n=10) according to the proposed design: CON (Control, no treatment or silane application), ABR+PS (Abrasion with diamond tip + application of Palabond commercial silane), JAT+PS (Blasting with 50μm aluminum oxide particles + application of Palabond commercial silane), ABR+ES (Abrasion with diamond tip + application of experimental silane added with thio-urethane), and JAT+ES (Blasting with 50μm aluminum oxide particles + application of experimental silane added with thio-urethane).

**Figure 1:**
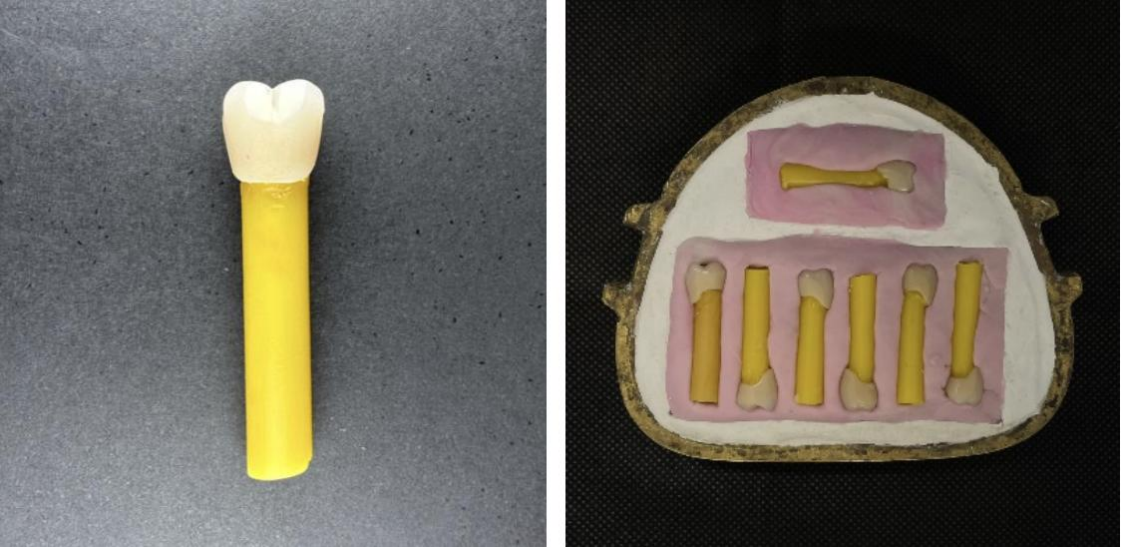
Tooth/wax stick set and inclusion in the metal flask.

Teeth were fixed to cylindrical wax sticks (Asper Chemical Industry; Sao Caetano do Sul, SP, Brazil) 50 mm long, and traditionally included in metal flasks (Safrany, Sao Paulo, SP, Brazil) according to the respective experimental treatments with Type III plaster coated with laboratory silicone (Zetalabor, Rovigo, Italy), as shown in Figure 1. Tooth/wax stick sets were removed from the flask,

the tooth separated from the wax stick, and the fitting area was cleaned with household detergent (Ypê; Química Amparo, Amparo, SP, Brazil) to eliminate wax residues.

### Preparation of the tooth fitting area

After preparation of the fitting area according to the following treatments: CON, ABR+PS, ABR+ES, JAT+PS, and JAT+ES, the teeth were brushed with a solution of water/household detergent (Ypê) and dried with air jets. The silanes were applied individually to the tooth fitting area with a micro dental brush (KG Sorensen, Serra, ES, Brazil) according to each experimental treatment.

For blasting, the area of the tooth fitting was blasted with aluminum oxide with 50-micrometer particles using an air jet device (BioArt; Dental Products, Sao Carlos, SP, Brazil). The aluminum oxide jet was aimed at the tooth at an angle of 45 degrees, 1cm away from the tooth for 5s. For abrasion, the tooth fitting area was lightly abraded with a PM 82 G cylindrical diamond drill (KG Sorensen; Serra, ES, Brazil) using a micromotor (Beltec; Araraquara, SP, Brazil), to remove the superficial glaze of the tooth fitting area, act with 5000 rpm for 5s.

### Application of commercial silane (Palabond)

In the ABR+PS and JAT+PS treatments, the Palabond commercial silane (Kulzer, Sao Paulo, SP, Brazil) was applied according to the manufacturer's instructions. Two applications were made with an interval of 30s between each application. After the second application, the thermo-activated acrylic resin mass (VIPI, Pirassununga, SP, Brazil) prepared according to the manufacturer's recommendations was placed in contact with the tooth fitting area filling the mold left by the wax stick.

### Application of experimental silane added with thio-urethane

The experimental silane incorporated with thio-urethane was applied in the ABR+ES and JAT+ES treatments. Two applications of the experimental silane were carried out using a micro dental brush (KG Sorensen). After the second application, the acrylic resin putty (VIPI) was applied as described in item 4.5.

The synthesis of the thio-urethane oligomer used in the study as an experimental silane occurred in the laboratories of Oregon Health and Science University (OHSU, Portland, USA), from the combination of thiol-pentaerythritol tetra-3-mercaptopropionate (PETMP) with isocyanate HDMI (4,4'-cyclic dicyclohexylmethane diisocyanate) ^22^.

### Polymerization of the acrylic resin

After inserting the teeth into the molds of the respective metal flasks and applying the acrylic resin in the plastic phase in accordance with the manufacturer's guidelines (VIPI), the tooth/resin sets were covered with a polyethylene film. The flask was subjected to controlled gradual compression in a hydraulic press (VH Press, EssenceDental, Araraquara, SP, Brazil) until it reached 900 kgf and then opened. After removing the plastic film and resin excess, the flask was closed again and subjected to final pressing (1,250 kgf). Before being transferred to the polymerization unit (Thermotron, Bass, Barueri, SP), the metal flask remained in the pressure clamp for 2h, as recommended by the acrylic resin manufacturer (VIPI). The polymerization of the acrylic resin occurred in a water bath heated to 74^o^C for 9h and the flask cooled at room temperature for 12h. After, the tooth/resin sets were removed from the flask.

**Figure 2:**
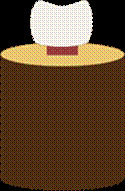
Schematic drawing of the tooth/resin set included in rigid PVC tube.

### Sample preparation for shear test

The tooth/resin sets were cleaned to eliminate residues of acrylic resin, inclusion plaster, and/or insulators. The sets were individually fixed by the resin rod in rigid PVC tubes with 50 mm in diameter using chemically activated acrylic resin (VIPI). To standardize the position of the sets during the mechanical shear test, a distance of 1 mm was established between the tooth and the surface of the rigid PVC cylinder (Figure 2).

### Shear test

**Figure 3:**
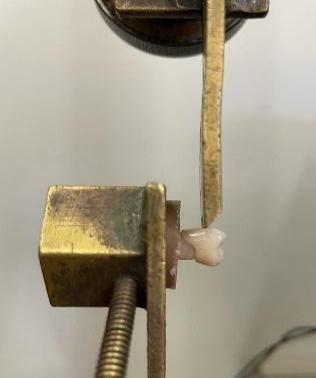
Position of the tooth/resin set for the shear bond test.

The tooth/resin sets were subjected to a shear strength test in a universal testing machine (Instron; Sao Jose dos Pinhais, PR). In this procedure, a metal rod with a chisel-shaped tip was positioned in the medial region of the buccal surface of the artificial tooth, applying compression force until the tooth/resin bond failed (Figure 3).

### Load unit considered in the shear bond test

During the development of the compression test, the measurement related to the machine peak load was carried out using two different units: Newton (N) and kilogram-force (Kgf). Although both units are used to quantify force, they are based on differing unit systems. The Newton is recognized as the unit of force in the International System of Units (SI), with a definition based on the fundamental units meter (m), kilogram (Kg), and second (s). The Newton unit represents the force required to propel 1 kg of mass at a speed of 1 meter/s^2^ (Meter/second squared). The kilogram-force unit (Kgf) belongs to the technical system of units and is related to the weight of 1 kg subject to the standard acceleration of gravity on the Earth’s surface, approximately 9.81 m/s² (meter per second squared).

### Representation of failure strength

Numerical values of failure strength are commonly expressed in Kg/cm² (Kilograms per centimeter squared) or MPa (Mega Pascal). These units represent measurements of compression or tension, reflecting the intensity of the force distributed per unit area. In the study of materials, these units serve to quantify the material resistance capacity related to shear force, preceding the moment of failure. The use of these measurements allows a detailed understanding and precise analysis of the structural performance of the materials evaluated, under specific conditions imposed by the test.

### Conversion from Kg/cm² to MPa

The conversion from Kg/cm² to MPa is based on the relationship between compression units in the metric system. Considering that 1 Pa (Pascal) is equal to 1 N/m² and that 1 Kgf is approximately equal to 9.81 N, the conversion Kg/cm² to MPa is the following:

1 Kg/cm² = 1 Kgf/cm² × 9.81 N/Kgf × 10.0001 cm²/m² = 0.0981 MPa

Therefore, to convert a failure strength value from Kg/cm² to MPa, the Kg/cm² value is multiplied by 0.0981.

### Statistical analysis

The shear strength data organized in an Excel spreadsheet were subjected to statistical analysis using SANEST (Statistical Analysis System). The microshear strength data were evaluated for normality, and subjected to one-way ANOVA (treatment), and Tukey's test with a significance level of 5%.

## Results

### Analysis of variance

The analysis of variance (ANOVA) showed statistically significant differences among treatments (p<0.00001), indicating that the treatment type showed a significant influence on the bond strength of the artificial tooth/acrylic resin for conventional complete denture base. The sum of squares between treatments was 724.1266502, while the sum of squares of the residual was 674.9937439, which highlights the variability attributed to the different treatments compared to intra-treatment variability (Table 1).

**Table 1:** Analysis of variance.

Variation	DF	SS	MS	F value	Prob.>F
Treatment	4	724.1266502	181.0316625	12.0689	0.00001
Residue		674.9937439	14.9998610		
Total	49	1399.1203941			

Average = 22.174601

Coefficient of variation = 17.466%

### Multiple comparisons of means

Tukey's test (5%) was applied to establish multiple comparisons between treatment-identified specific differences (Table 2). The JAT+PS promoted the highest strength (28.39±5.52), significantly higher than other treatments, followed by ABR+ES (24.35±2.42), while ABR+PS (21.20±2.97) was intermediate. JAT+ES (18.00±4.32) and CON (18.91±3.31) were statistically similar with lower values compared to the other treatments.

**Table 2:** Tukey’s test for treatment mean.

	Mean (SD)	5%
JAT+PS	28.39 (5.52)	a
ABR+ES	24.35 (2.42)	b
AB+PS	21.20 (2.97)	bc
JAT+ES	18.00 (4.32)	c
CON	18.91 (3.31)	c

Means followed by different letters differ from each other at the indicated significant level. D.M.S. 5% = 4.92958 - D.M.S. 1% = 6.00428

## Discussion

Complete dentures manufactured with acrylic resins associated with artificial teeth is yet recommended for rehabilitation of partially or completely edentulous patients. Acrylic resin has been indicated for several decades and when used properly provides reliable and affordable dental rehabilitation for most patients.

Partial or complete dentures are commonly constructed for the elderly population. However, tooth debonding from the complete dentures can be frustrating to the patients as well as for the dentist. Therefore, the selection of compatible combinations of denture base resin and acrylic tooth may reduce the number of prosthesis failures and the resultant repairs ^1^.

The difficulty in establishing lasting bond between artificial tooth/ denture base is a challenge during the manufacturing of this prosthesis type. Thus, a study showed that the failure of the bond between tooth/acrylic resin base is the cause of the tooth debonding causing inconveniences and costly repair. The optimal combination of denture tooth, denture base material, and processing method has not yet totally been established ^2^.

In the current study, it was evaluated whether the application of experimental silane incorporated with thio-urethane could improve the bond strength between artificial tooth/acrylic resin for conventional complete denture bases, regardless of the treatment of the artificial tooth fitting area. However, the results showed a statistically significant difference (p<0.00001), showing that the treatments promoted significant influence on the bond strength between tooth/acrylic resin for denture base. On the other hand, the JAT+ES treatment showed a value statistically similar to the CON; therefore, opposing the hypothesis formulated that the experimental silane incorporated with thio-urethane would increase the bond strength between artificial tooth/acrylic resin whatever the treatment of the tooth fitting area.

A previous study showed that blasting with Al₂O₃ particles was more effective in increasing the bond strength between artificial tooth/acrylic resin, regardless of whether it is applied alone or combined with other treatment types. However, there was greater bond strength when the blasting was associated to application of the PMMA monomer ^23^.

The current study partially confirms the positive influence of the results obtained with Al₂O₃ particles blasting, considering that the JAT+PS treatment consisting of blasting + Palabond silane exhibited the highest bond strength. The Palabond bonding agent contains methacrylic acid, dimethacrylate, and methylmethacrylate, components similar to those of the PMMA monomer. However, the JAT+ES treatment combining blasting + experimental silane did not promote a significant improvement in bond strength when compared to CON (Control).

However, another study showed that abrasion of the tooth fitting area with a diamond tip significantly increased the bond strength of the tooth/acrylic resin interface compared to the Al₂O₃ particles blasting with lower adhesion strength ^24^. These results show a divergence with the data of a previous study ^23^, and with the results of the current study.

Other previous works also showed that monomers used for chemical treatment of the fitting area of the artificial tooth associated with efficient wax removal methods can significantly improve the strength of the tooth/acrylic resin bond ^5^ ^,^ ^25^ ^,^ ^26^. The current study appears to confirm the claims of these aforementioned studies since the artificial tooth without wax residue associated with the Palabond commercial silane promoted greater bond strength for both treatments (JAT+PS and ABR+PS) compared to the control (CON).

Furthermore, other chemical treatments and different acrylic resins also showed different strength values of the tooth/resin bond. Acrylic tooth bonded to high-impact denture base materials (DPI Tuff and Trevalon HI) with different treatments at the ridge lap area of the tooth showed that the dichloromethane/monomer mixture combined with Trevalon HI high-impact resin showed the highest bond strength ^26^, and the addition of monomers to the tooth surface significantly strengthened the shear bonding of acrylic base resin to the tooth ^27^ ^,^ ^28^.

Air abrasion (Alumina-blasting) and dichloromethane application improved the shear bond strength for acrylic tooth/heat-polymerized denture base resins compared to the untreated group. However, none of the surface treatments showed significant improvement with the CAD/CAM denture base resin. All surface treatments reduced the shear bond strength for the CAD/CAM resin tooth, while air abrasion only increased the bond strength with heat-polymerized resin ^29^. In addition, for Lucitone 199 acrylic resin, the highest failure resulted when the tooth ridge lap was left with the intact glaze, without any mechanical means of attachment of the tooth to the denture base, a similar result compared to monomer application. For Ivocap resin, the highest failure load resulted when the tooth ridge lap had retention but did not have monomer treatment and no glaze significant influence ^15^.

The study evaluated the influence of surface treatments of the ridge lap area of PMMA denture tooth on the shear bond strength with different resins, being a specially designed resin for digital denture, a common self-curing resin for repairs, and a conventional heat-curing resin, according to three surface conditionings (no treatment, sandblasting or sandblasting plus methyl-methacrylate application). The results showed that there was a statistically significant interaction among resins and tooth treatments. The surface treatment seems not to affect the tooth/base bond; however, the tooth bonded to a CAD/CAM denture base using PMMA self-curing resin for repairs showed the highest bond strength compared to other treatments ^16^.

The thio-urethane oligomers incorporation has shown promising improvement in the mechanical properties of several resin dental materials. Double-activation composite cement added with thio-urethane showed greater mechanical strength, fracture toughness, and bond strength to dentin, as well as reduced contraction stress ^17^. Another study showed that thio-urethane oligomers added to composite resin increased the biaxial flexural strength of the ceramic/composite resin and the dentin/zirconia micro tensile bond strength ^19^, as well as thio-urethane oligomers were able to increase the µTBS of composite/cement/ceramic samples; however, thermal/mechanical cycling reduced µTBS for all resin cement ^30^. Furthermore, experimental silane added with thio-urethane applied to fiberglass posts promoted different values of bond strength to the root thirds, with improved results compared to the control ^20^. The addition of thio-urethane in the matrix of experimental adhesives reduced the mechanical properties after storage in water; however, the bond strength was not influenced after 24h or 6 months of water aging ^31^.

The literature has shown the effect of the thio-urethane incorporated into various dental materials, such as ceramic, adhesive, composite, and fiberglass posts. However, it only lists one study on the mechanical properties of acrylic resin incorporated with different thio-urethane oligomers. This study verified the potential toughening of acrylic resin polymerized by the microwave energy showing that, except in the dimensional stability, other properties promoted lower or similar results compared to the control, depending on the concentration and thio-urethane type incorporated into acrylic resin ^21^.

In the current study, considerable improvement in the strength of the tooth/acrylic resin bond was observed when the tooth fitting area was subjected to blasting with Al₂O₃ particles + application of the Palabond silane (JAT+PS). It was not possible to determine why there was no significant improvement when abrasion with diamond tip + Palabond silane (ABR+PS) was used. On the other hand, considerable improvement in the bond strength was shown when the tooth fitting area was subjected to diamond tip treatment + experimental silane (ABR+ES) compared to blasting with Al₂O₃ particles + application of the experimental silane (JAT+ES), with the result similar to Control (CON).

 In general, the determining factor in the tooth/resin base bond strength is the difference in the morphology of the teeth fixation area promoted by abrasive treatments (diamond tip or blasting with Al₂O₃ particles). Although the current study has not evaluated the roughness promoted in the tooth fitting area by abrasion with diamond tip or blasting with Al₂O₃ particles, a previous study showed that the modification of the tooth fitting area by grinding or grooving did not provide a significant difference compared to unmodified surfaces ^5^. In addition, the strength of sandblasted teeth bonded to heat-polymerized denture base was significantly higher than those with an abradded surface and the control. However, the investigation showed that combined cohesive failures (tooth and denture base) occurred in all treatments ^32^.

Furthermore, the lack of comparison between the teeth surfaces treated with diamond tip abrasion or blasting with Al₂O₃ particles in the current investigation can be considered a limitation of the study.

## Conclusion

Regardless of the study's limitation, the following conclusions can be considered: The artificial teeth treatments with experimental silane incorporated with thio-urethane associated with blasting or abrasion promoted different strength values when bonded to acrylic resin for denture base.
